# 

**Published:** 1897-08

**Authors:** 


### CONTENTS.

COMMUNICATIONS.

Tumors of the Maxilla.......... 365
Resection and Reproduction of the Maxillae.......... 373
Chairman's Address.......... 377
Cataphoresis vs. The Direct Application of the Galvanic Current for Obtunding Sensitive Dentine; and How to Conduct a Practice that Excludes Both.......... 381
A Series of Clinical Cases.......... 384
Pathologic Conditions of the Pharynx and Contiguous Structures During Early Childhood—Prime Factors in the Etiology of Mal-Formed Maxillæ and Irregular Teeth, Etc.......... 387
A Glance at Some Relations of Dentistry to General Medicine.......... 389
Anterior Pillars of the Fauces—Etiology and Treatment.......... 391
The Need of Dental Instruction in Medical Schools.......... 392
The Implantation of Sterilized Roots of Animals' Teeth, Carrying Artificial Crowns.......... 394
Hyperæmia and Inflammation of the Dental Pulp.......... 395

SELECTIONS.

Through the Telephone.......... 393
A Plea for a Greater Use of Non-Cohesive Gold.......... 397
Medical Education.......... 399
Silver as an Antiseptic, from a Surgical and Therapeutic Viewpoint 400
The Medicine Habit.......... 401
The Office Hour.......... 402
Humbugging in Medicine.......... 403
Remarks on the Treatment of Fractures.......... 404
Then and Now.......... 405
Report of the Commissioner of Education for the Year 1894–95.......... 406
A Word to the Wise is Sufficient.......... 407
Full Deep Breathing.......... 416

COMMENCEMENTS.

Chicago College of Dental Surgery.......... 407
Philadelphia Dental College.......... 408
Pennsylvania College of Dental Surgery.......... 410
State University of Iowa.......... 411
Tennessee Medical College, Knoxville, Dental Department.......... 411
Dental Department of Harvard University.......... 411
Dental Department of University of Tennessee.......... 412

NOTICES.

Southwestern Michigan and Northern Indiana Dental Society.......... 412
The Executive Committee of the American Dental Society of Europe.......... 412
Northern Iowa Dental Society.......... 413

EDITORIAL

Vacation.......... 413
Cleanliness.......... 415

